# Mechanisms of Resistance and Current Treatment Options for Glioblastoma Multiforme (GBM)

**DOI:** 10.3390/cancers15072116

**Published:** 2023-04-01

**Authors:** Satya Siva Kishan Yalamarty, Nina Filipczak, Xiang Li, Md Abdus Subhan, Farzana Parveen, Janaína Artem Ataide, Bharat Ashok Rajmalani, Vladimir P. Torchilin

**Affiliations:** 1 Center for Pharmaceutical Biotechnology and Nanomedicine (CPBN), Department of Pharmaceutical Sciences, Northeastern University, Boston, MA 02115, USA; 2 State Key Laboratory of Innovative Drug and Efficient Energy-Saving Pharmaceutical Equipment, Jiangxi University of Chinese Medicine, Nanchang 330006, China; 3 Department of Chemistry, ShahJalal University of Science and Technology, Sylhet 3114, Bangladesh; 4 Department of Pharmaceutics, Faculty of Pharmacy, The Islamia University of Bahawalpur, Bahawalpur 63100, Pakistan; 5 Department of Pharmacy Services, DHQ Hospital, Jhang 35200, Pakistan; 6 Faculty of Pharmaceutical Sciences, University of Campinas (UNICAMP), Campinas 13083-871, Brazil; 7 Department of Chemical Engineering, Northeastern University, Boston, MA 02115, USA

**Keywords:** glioblastoma multiforme, nanomedicine, drug delivery systems, resistance, immunotherapy

## Abstract

**Simple Summary:**

Glioblastoma multiforme is one of the hardest-to-treat brain tumors, often resistant to conventional treatments such as chemotherapy and radiation therapy. Despite advances in cancer research and treatment, the survival rate for glioblastoma patients remains low, with most patients only surviving for a few months to a year after diagnosis. This is due in part to the nature of glioblastoma tumors, which contain cells that are highly resistant to traditional cancer treatments. These cancer cells can evade the effects of chemotherapy and radiation therapy, making it difficult to achieve lasting remissions or cures. This work aims to summarize recent findings about this tumor and the progress in developing new treatment options. The highest focus of this paper is on the mechanisms of glioblastoma multiforme resistance and the possibility to reverse it using nanocarriers.

**Abstract:**

Glioblastoma multiforme (GBM) is a highly aggressive form of brain cancer that is difficult to treat due to its resistance to both radiation and chemotherapy. This resistance is largely due to the unique biology of GBM cells, which can evade the effects of conventional treatments through mechanisms such as increased resistance to cell death and rapid regeneration of cancerous cells. Additionally, the blood–brain barrier makes it difficult for chemotherapy drugs to reach GBM cells, leading to reduced effectiveness. Despite these challenges, there are several treatment options available for GBM. The standard of care for newly diagnosed GBM patients involves surgical resection followed by concurrent chemoradiotherapy and adjuvant chemotherapy. Emerging treatments include immunotherapy, such as checkpoint inhibitors, and targeted therapies, such as bevacizumab, that attempt to attack specific vulnerabilities in GBM cells. Another promising approach is the use of tumor-treating fields, a type of electric field therapy that has been shown to slow the growth of GBM cells. Clinical trials are ongoing to evaluate the safety and efficacy of these and other innovative treatments for GBM, intending to improve with outcomes for patients.

## 1. Introduction

Neoplasms in the central nervous system (CNS) arise from various types of cells and make up about 2% of all cancers. About 95% of malignant CNS tumors occur in the brain, with 75% of them arising from glial cells, also known as gliomas. Glioblastoma multiforme (GBM) accounts for over 50% of all gliomas. According to the WHO, gliomas are divided into grades I–IV, which specifies the different degree of malignancy [1]. Glioblastoma multiforme is considered a type IV glioma. It is the most common and malignant, with an average five-year survival rate of 7.2% and an average length of survival 15 months [2]. In glioma type I, pilocytic astrocytoma and mutation in the neurofibromin I (NF I) gene are the most common features [1]. Grade II and III gliomas occur due to mutations in TP53 and α-thalassemia/mental retardation syndrome X-linked (ATRX) gene. Grades II and III are rare in children and commonly manifest in young adults. Children usually do not have IDH mutations and Ip/19q co-deletion [3,4,5]. WHO grade IV glioma is commonly seen in patients over 50 years. There is growing evidence that glioblastomas can also develop in children, adolescents, and young adults and that these tumors are genetically unique from those found in older adults [6].

Glioblastoma multiforme is typically seen in older adults above 60 years of age. It accounts for 45.2% of CNS and malignant tumors in adults [7]. In the United States, the incidence rate is only 3.19 in 100,000, which is very rare. In pediatric patients, the incidence rate is 0.85 per 100,000. The male–female incidence ratio ranges from 1.2 to 2.6, which shows it is more prevalent in men than women. According to the study, 83% of the patients were more than 50 years of age, and 47.9% were above 65 years old. Age plays a vital role in mortality risk factors in children, with a 6–10-year hazard ratio of 1.408 and an 11–19-year age hazard ratio of 1.406. The survival rate of patients under 50 is 8.8 months, and the survival rate of patients above 50 is 4.55 months [8]. TERT and TP53 promoter mutations are related to the age of the patients. They are frequently seen in older patients, while the opposite is true for IDH1 mutations [9].

The etiology of glioblastoma has not yet been fully discovered. A high dose of ionizing radiation is a risk factor for developing GBM [10]. More than 116 cases of GBM linked to radiation exposure have been documented since the 1960s. It has been predicted, computed, or estimated that the overall probability of developing GBM after radiotherapy is 2.5% [2,10]. Lower doses of radiation used to treat skin hemangioma and capitis in infants are also associated with developing type 4 gliomas. There are significantly fewer data available regarding the development of GBM in adults. Many studies of the Japanese population exposed to atomic bomb irradiation in Nagasaki and Hiroshima showed increased gliomas of all types [11]. Environmental variables, including smoking, nutritional risk factors, electromagnetic fields from cell phones, severe head injuries, occupational risk factors, and pesticide exposure, have not been conclusively linked to GBM [12]. According to a few studies, the development of GBM may be influenced by ovarian steroid hormones [13]. Additionally, it has been suggested that allergies and infections protect against GBM by activating immune surveillance mechanisms. According to a 2007 meta-analysis, those with allergies have a 40% lower risk of acquiring gliomas than those without allergies. Additionally, it has been discovered that gliomas run in families, but the susceptibility gene is still unknown. Only 5–10% of instances with a genetic predisposition have been identified [14].

GBM can be divided into primary and secondary according to their clinical characteristics. The EGFR gene mutation and amplification, the overexpression of mouse double minute 2 (MDM2), the deletion of p16, the loss of heterozygosity (LOH) of chromosome 10q harboring phosphatase and tensin homolog (PTEN) [15], and the mutation of the TERT promoter are all hallmark abnormalities of primary GBM. Platelet-derived growth factor A and platelet-derived growth factor receptor alpha (PDGFA/PDGFRa) overexpression, retinoblastoma (RB), LOH of 19q, and mutations in IDH1/2, TP53, and ATRX are some of the distinguishing characteristics of secondary GBMs [16]. Recent studies in pediatric patients have suggested that there might be a 3rd major category of GBM which is distinct from the two GBMs on basis of histone H3F3 gene mutations [16,17].

## 2. Conventional Treatment Options

**Figure 1:**
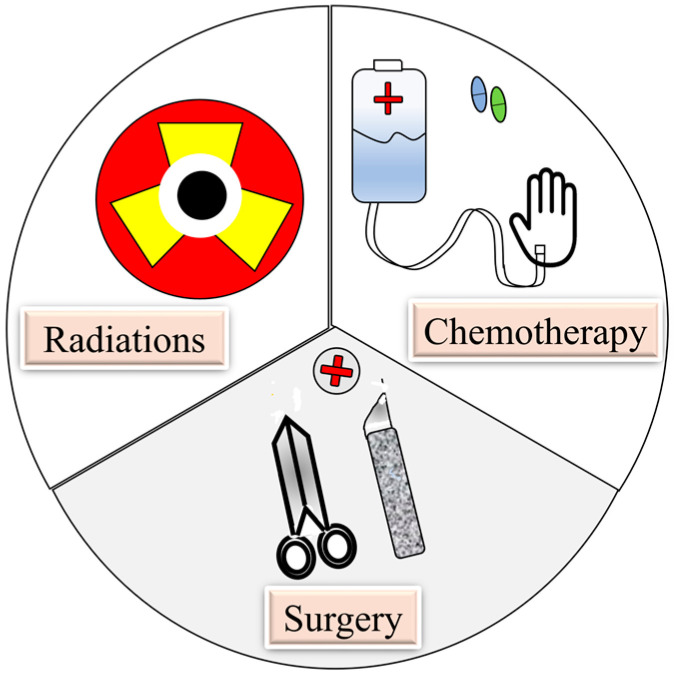
Traditional therapies for GBM.

The classical traditions to treat GBM mainly relied on surgery, chemotherapy, and radiotherapy, which are routinely used in clinics (Figure 1).

### 2.1. Traditional Therapy

#### 2.1.1. Surgical Resection

Scientists started to work on the removal of brain tumors by using surgical resections in the early 1980s, and with the development of frameless stereotaxy in the 1990s, the field of surgical resection was revolutionized. The use of image guidance enables the precise placement of surgical instruments. After the development of many modern imaging technologies, this field has been modernized [18].

The removal of brain tumors became easier after the development of the “brain mapping” technique, in which cerebral cortical stimulation facilitates the localization of the cortex region of the brain to be avoided during brain surgery. The term gross total resection (GTR) is widely used in brain tumor surgery to indicate the extent of brain tumor resection. It is directly correlated with the survival time of GBM patients due to the various challenges involved [19,20]. Successful tumor site identification and avoiding the adjacent area of the tumor during surgery are major aspects of consideration. The tumor margins are roughly determined even with intra-operative imaging technologies and finger-like projections that are visible even with a microscope [21].

The development of new and more sensitive imaging techniques is a very good tool for GBM surgery. These techniques help in more precise biopsy of tumor cells. These techniques also help in the identification of tumor margins. The two most used imaging techniques are computed tomography (CT) and MRI. CT is a more emergent technique than MRI, which is quite old and a gold standard technique in the surgery of brain tumors because of its high soft tissue contrast and high resolution. Because of its high resolution, many features of the tumor can be clarified such as tumor margins, blood vessels in the tumor, necrosis, hemorrhage, and blood perfusion [22,23,24].

The intra-operative fluorescence imaging and MRI can be helpful to cope with the challenges of “brain shift” which causes discrepancies during brain tumor surgery by creating hurdles in locating the exact position of the tumor. It may cause discrepancies between the locations of the tumor and critical brain structures in preoperative imaging studies and the operating room. Some shifts can be up to 2 cm in distance and result from causes that may be physical (patient position or gravity), surgical (type of equipment used, tissue/fluid loss during the procedure), or biological (tumor type or location, and drugs used to manage intracranial pressure). The increase in the duration of the surgery may intensify these shifts that cannot be corrected by using neuronavigational devices to derive stereotaxic capabilities from pre-operative MRI images [25].

The fluorescent imaging technique has been used historically in brain surgery that is performed with fluorescein, indocyanine green (ICG), and 5-aminolevulinic acid (5-ALA). This technique is one of the first brain imaging methods that does not cross the blood–brain barrier. It only penetrates in the area of high permeability having high-grade gliomas [26,27].

Intra-operative MRI and ultrasound during brain surgery help to locate the residual cancer cells. In this method, the surgeon surgically removes the tumor and then performs MRI in the operation room, and if any residual tissue is found, then this residual cancer tissue is also removed in the same surgical procedure [28,29].

#### 2.1.2. Cytotoxic and Anti-Angiogenic Chemotherapy

The standard post-surgical treatment of GBM includes 6 weeks of concomitant temozolomide (TMZ) (75 mg/m^2^) and radiation therapy, followed by adjuvant TMZ (150–200 mg/m^2^) for 5 days every 28 days for six cycles. TMZ is the most commonly used chemotherapeutic drug in the post-surgical treatment of GBM, and it directly damages the tumor cells by methylation of the purine base of tumor cells [30,31]. The main cytotoxic action of TMZ is due to the formation of 6-methylguanine lesions which causes apoptosis, autophagy, and cellular senescence of tumor cells [32]. The radiation-sensitizing properties of this drug also increase the radiation-induced cell death of cancer cells when administered in combination with radiation therapy. The major side effects associated with the TMZ treatment regimen include hematologic toxicity and thrombocytopenia, which has been reported in 10–20% of patients in phase 2 clinical trials [33,34,35].

Several nitrosourea drugs have also been tested for the treatment of brain tumors. Carmustine is a small nitrogen mustard agent that causes the inter-stand cross-linkage between cytosine and guanine base in tumor cell DNA. FDA-approved biodegradable discs of carmustine are available, and they are placed in the resection cavity during surgery, releasing the drug slowly over two weeks. Alkyl guanine transferase (AGT) can reverse the alkylating therapeutic effect of carmustine and AGT inhibitors are used in combination for better therapeutic effects. Intracranial infection and abnormal wound healing may occur in this treatment regimen [36,37,38,39,40]. Lomustine and Fotemustine are other important alkylating agents used orally in the treatment of recurrent GBM that can cross the blood–brain barrier due to small size and high lipophilicity [41,42,43].

Bevacizumab is an intravenous preparation of anti-angiogenic humanized monoclonal antibody used to cure recurrent GBM. The vascular endothelial growth factor A (VEGF-A) is a potential target of bevacizumab. Receptor binding initiates the formation of new blood vessels, and its level increases up to 30 times its normal level in the case of GBM. It is evident from many studies that progression-free survival has been improved significantly in patients using bevacizumab, but it does not significantly improve the overall survival rate of newly diagnosed patients with GBM. The most common side effect associated with the use of bevacizumab is hypertension and leukopenia [44,45].

#### 2.1.3. Radiation Therapy (RT)

Radiation in the form of X-ray photons, gamma photons, and protons are emerging tools to provide local control for microscopic cancer cells, which cannot be treated by surgical resection. Radiation therapy (RT) is commonly administered over 6 weeks in two gray fractions with a dose of 40–60 gray by using a specific device with multi-leaf collimators to deliver a very sharp beam of radiation to target cells allowing small (1–2 cm) margin of radiation in the periphery of a tumor [46]. In three-dimensional conformal radiation therapy (3D-CRT), 3D X-rays are used to target the microscopic tumor cells that cannot be addressed by surgery. X-rays are delivered to target tumor cells with the aid of CT scans and MRI imaging from different angles. Targeting of a tumor with X-rays causes low linear energy transfer that results in both direct (one-third of total treatment effect) and indirect (two-thirds of total treatment effect) damage of DNA. Conformal RT is a commonly used technique with fewer side effects to treat residual GBM cells that targets only cancer cells and has no effect on normal cells. This technique is more complex to some extent because of the use of multi-collimated beams to avoid injury to critical structures such as the cornea, optical nerves, and brainstem [47,48].

Another form of RT is stereotaxic radiosurgery (SRS), which can deliver even larger doses of radiation on target cells by using nonparallel converging radiation beams. The dose of radiation is given in lesser fractions (1–5) at a higher dose of 15+ gray. Gamma rays are delivered to targets from a cobalt source. Due to the heat transfer properties of gamma rays, the radiation can be delivered very specifically to target cells without affecting normal cells. SRS can be used in the treatment of brain metastasis, mainly to avoid the side effects which are associated with whole-brain RT [49,50,51]. Research is being conducted on another RT technique called brachytherapy, which involves the implementation of one or more radioactive vectors into the tumor bed during the surgery. The common isotopes used in this method are iodine-125 and iridium-192. Brachytherapy is associated with many side effects which include necrosis, vascular injury, and radiation exposure to those in close contact with other people [52,53].

Combination therapies are more successful in treating GBM such as the use of carbon proton irradiation in combination with TMZ to increase the overall survival of GBM patients as compared to the patient undergoing the treatment with the combination of TMZ and photon-induced irradiation. An important obstruction to effective RT is a hypoxic environment in which oxygen increases the response of cells to low linear energy transfer radiation. The irradiation of tissues to form DNA radicals creates an environment to react with oxygen, causing permanent cell damage. In hypoxic tumor environments, the damaged DNA has added time to repair and reduce radiation injury. It has been proved that hypoxic tissue requires an approximately three-times greater radiation dose as compared to non-hypoxic tissue to achieve the same therapeutic benefit [47,54,55].

### 2.2. Mechanisms of Resistance

#### 2.2.1. DNA-Repair Enzymes

The cytotoxicity of DNA after O6-methylation of guanine adducts (O6-MeG) and cell death by specific action of TMZ during progression is inhibited when DNA repair enzyme O6-methylguanine-DNA methyltransferase (MGMT) prohibits this process to develop resistance. This mechanism is the main cause of drug resistance in the therapy of recurrent GBM (up to 75%). The level of MGMT is directly associated with the efficacy of alkylating drugs on cancer cells in brain tumors [56,57,58,59,60].

#### 2.2.2. Mismatch-Repair (MMR) Complex Formation

When the MGMT enzyme is not present to remove the methyl group in the DNA replication process, thymine is mistakenly inserted at O6-MeG by the DNA polymerase enzyme. This O6-MeG–T mismatch is called MMR complex. The main cytotoxic agent for DNA replication in tumor cells is O6-methyl guanine produced by alkylating drugs. This cytotoxic product can only be removed by MGMT enzyme or by the deficiency of MMR. The deficiency of MMR refers to the strong drug resistance to alkylating drugs such as TMZ, procarbazine, and N-methyl-N’-nitro-N-nitrosoguanidine (MNNG) [61,62].

#### 2.2.3. Glioma Initiating Cells (GICs)

GICs are cancer stem cells having specific properties that support tumor development, recurrences, and therapeutic resistance in the therapy of GBM. These cells have self-renewal and tumorigenic properties. For the first time, Singh et al. described the population of tumor CD133+ cells as GICs that initiate the GBM growth in non-obese diabetic severe combined immunodeficient mice. CD133 is the main marker of GICs, but these cells may also be present in normal stem cells. So, other multiple markers (CD44 and ATP binding cassette transporters) are also needed for the characterization of GICs [63,64,65].

GICs can show sensitizing mechanisms to radiation therapy. The “checkpoint kinases” (Chk1 and Chk2) play a pivotal role in the cell division cycle. Chk1 is activated by the signal received in response to DNA damage. This enzyme will delay the cell division in the G2 phase until the DNA repair is completed. Similarly, the Chk2 enzyme is activated after the double standard break in DNA and saves the cells to divide in an uncontrolled manner. The presence of these enzymes results in decreased sensitivity towards radiation therapy and thus resistance to the radiation. It has been described by Bao et al. that the addition of inhibitors to these enzymes in GBM therapy enhances the radioactivity of GICs [66].

ATP Binding Cassette (ABC) Transporters are the most common and largest family of trans-membrane protein pumps that are involved in the movement of cholesterol, bile acids, ions, and peptides across the cell membrane. In normal cells, ABC transporters are inactive but in tumor cells, these transporters are highly active and overexpressed causing hindrances in the drug delivery across the membrane [67]. ABC transporters exhibit therapy resistance by promoting the efflux of exogenous agents, such as TMZ, at cellular and blood–brain barrier levels. These transporters have 49 members and 9 subfamilies out of which ABCB1 (MDR1), ABCC1 (MRP1), and ABCG2 (BCRP1) are well-known ABC transporters that have been found in tumor cells. ABBC1 is one of the most important ABC transporters that are involved in the recurrence of GBM. GICs are present in large quantities in hypoxic tumor cells. Less oxygen results in the expression of ABCC1 and ABCB1 and therefore leads to resistance to chemotherapy [68,69].

#### 2.2.4. Hypoxia and Autophagy

Tumor hypoxia is one of the major reasons for the poor prognosis of many cancer types such as GBM. The less oxygenated area may lead to the development of GICs that trigger the activation of tumor genesis pathways and resistance to tumor therapy. Hypoxia-inducible factor 1 (HIF-1) is an important factor involved in the promotion of the tumorigenic activity. In cancer cells, micro-vascular thrombosis also increases hypoxic conditions. GICs are mostly present in the tumor cell areas where oxygen is less (1.25% O_2_) than in pre-tumor cell areas (2.5% O_2_). Overall tumor volume and GIC development are inversely proportional to the oxygen tension [70,71,72].

It is evident from many studies that indicate autophagy is a result of a hypoxic environment in the cells as a cytoprotective mechanism. It is a cell-protective phenomenon that helps to clear the damaged proteins and other organelles for the maintenance of the homogenetic environment in the cell. Some metabolic precursors (amino acids, lipids) are also produced by autophagy that further support the cell metabolism process. In brain tumor cells, the autophagy process is activated by the GICs, which results in resistance to chemotherapies [38,73]. The sensitivity of GBM cells towards TMZ can be increased by the inhibition of the autophagy process. Chloroquine is an autophagy inhibitor agent that can be used to reduce the resistance of tumor cells toward anticancer therapy [71]. The patients treated with chemotherapy in combination with chloroquine had shown better survival in phase 3 clinical trials [74,75].

## 3. Treatment Challenges

Heterogeneity is a problem in cancers and GBM is recognized as a highly heterogenic cancer. Moreover, there are several challenges in treating GBM and delivering drugs to the site of action. This section illustrates some of the many challenges to treatment of GBM.

### 3.1. Blood–Brain Barrier (BBB)

**Figure 2:**
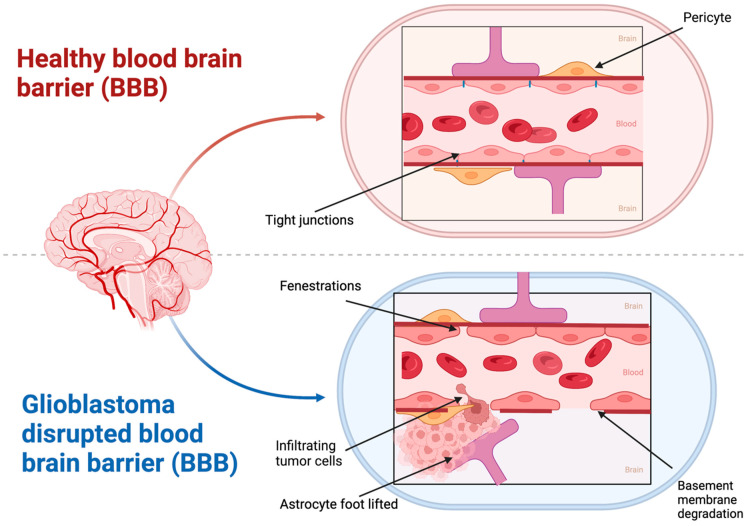
The depiction of the characteristic of a healthy BBB versus a disrupted BBB in GBM (created by Bio Render).

The outermost lining of the blood vessels in the brain and spinal cord comprises the blood–brain barrier (BBB). The barrier is a key immunological feature of the human body comprising several cells and that act as a structural and functional roadblock to any microorganisms in the bloodstream. A healthy BBB possesses tight junctions comprising astrocytes and pericytes. This feature helps in the selective passage of oxygen and nutrients into the central nervous system (CNS) thereby maintaining homeostasis (Figure 2) [76,77,78,79]. The BBB also inhibits the entry of drugs into the brain with the aid of resistance proteins such as P-glycoprotein (P-gp) and multidrug resistance proteins (MDRPs). These proteins not only prevent the entry of drugs into the brain but also reduce drug accumulation in the brain, thereby rendering therapy ineffective [30,80,81]. In conditions such as GBM, the BBB is disrupted and shows extensive infiltration. A series of steps are involved in the development of GBM, namely, migration of GBM cells into their surrounding vasculature, removal of astrocytic end foot processes, and later disruption of normal contact between endothelial cells and basement membrane by the secretion of glioma-derived factors [82]. Matrix metalloproteinases (MMPs) are induced by glioma-derived factors such as reactive oxygen species (ROS), transforming growth factor beta2 (TGF-β2), and caveolin1. The induction of MMPs cause disruption of the tight junctions between the endothelial cells [83]. This results in the formation of abnormal blood vessels by degradation of vessel basement membrane and surrounding extracellular matrix (ECM) and the migration of endothelial cells. Another reason new blood vessels are formed is the overexpression of vascular endothelial growth factor (VEGF), which can act as a hypoxia-inducible factor [84]. This rapid growth of a multilayered vasculature reflects the disruption of BBB, since tight junctions are disrupted by the genesis of these vasculatures. The disruption of BBB can be visualized with the help of a T1 gadolinium contrast agent in an MRI. The contrast agent accumulates only in the disrupted region and not in the intact region. Another interesting fact is that the BBB is disrupted at the primary tumor site and not at metastatic sites a few centimeters from the visible tumor. This is the main reason for GBM recurrences at a few centimeters from the surgical resection cavity [85,86]. Patients with GBM have a variable and heterogenous BBB disruption and intact BBB regions. This is sufficient to limit a drug’s passage through the barrier and reach the tumor site [87,88]. Vasogenic brain edema results in increased intracranial pressure with BBB leakage. This is a major clinical complication associated with GBM [84]. Passive drug diffusion is also lowered due to this increased intracranial pressure. These conditions of poor blood perfusion, high intratumoral interstitial fluid, variable BBB disruption, and active drug resistance mechanisms result in poor therapeutic efficacy in GBM [89].

### 3.2. Blood–Brain Tumor Barrier (BBTB)

The BBTB is a specialized barrier that separates the brain from the bloodstream and prevents the entry of most drugs into the brain. This makes it difficult to deliver therapeutic agents to the site of the tumor and effectively treat a cancer. Several approaches have been tried to overcome the challenges posed by BBTB in GBM treatment. These include developing new drugs that can penetrate the BBTB, using drug delivery systems that can transport drugs across the BBTB, and surgical removal of the BBTB to allow for direct drug administration to the tumor site. However, these approaches have yet to yield a cure for GBM, and more research is needed to fully understand BBTB and develop effective strategies for drug delivery. Recently, a study has shown that nanocarriers can be used to increase the penetration of drugs across the BBTB. However, the long-term effects and efficacy of these nanocarriers in treating GBM still need to be evaluated [90]. Additional research will likely lead to new and improved treatments for GBM that overcome the challenges posed by the BBTB.

### 3.3. Intra-Brain Tissue Diffusion

Intra-brain tissue diffusion is a major challenge in the treatment of glioblastoma multiforme (GBM), as the dense network of blood vessels, cells, and extracellular matrix within the brain can limit the diffusion of drugs. This can reduce the efficacy of treatment, as drugs may not reach all parts of a tumor. In addition, the invasive nature of GBM makes it difficult for drugs to reach all areas of the tumor, as the cancer often spreads throughout the brain [91].

Several strategies have been proposed to improve drug diffusion within the brain tissue. One approach involves the use of permeabilizing agents, which can temporarily increase the permeability of the blood vessels and allow for better drug diffusion. Another approach is the use of nanocarriers, such as liposomes or polymeric nanoparticles, to deliver drugs directly to the tumor site. These nanocarriers can improve the diffusion of drugs within the brain tissue and increase the efficacy of treatment [92]. Despite these advances, there are still many challenges to overcome to effectively treat GBM with drug delivery. Further research is needed to understand the factors that influence drug diffusion within the brain tissue and to develop more effective strategies for drug delivery. This could include the development of new nanocarriers, the use of combination therapy to enhance drug diffusion, and the optimization of drug delivery protocols to maximize the efficacy of treatment. In addition, it is important to consider the potential side effects of these strategies and to carefully evaluate the safety and efficacy of new treatments before they can be used in clinical practice.

### 3.4. Chemo-Radiation Resistance of Cancer Stem Cells (CSCs) in GBM

CSCs represent a small population of cells within a tumor that can self-renew and differentiate into various cell types, leading to tumor growth and recurrence. This resistance to conventional treatments is due to several factors, including the expression of drug efflux transporters, activation of DNA repair mechanisms, and the regulation of cell death pathways. One of the biggest challenges in the delivery of drugs to GBM CSCs is their location within the brain, which is protected by the blood–brain barrier (BBB). This presents a significant challenge for the effective delivery of chemotherapy drugs and other therapeutic agents to the site of the tumor [93]. To overcome this obstacle, several drug delivery strategies have been developed, including convection-enhanced delivery, implantable pumps, and focused ultrasound-mediated drug delivery.

Additionally, the unique biological characteristics of CSCs, such as their high resistance to apoptosis, their ability to form cancer spheres, and their expression of drug efflux transporters, have made it difficult to effectively target these cells with conventional chemotherapy drugs [94]. To overcome this, exploration of novel drug delivery strategies, including nanotechnology-based approaches, that are designed to specifically target CSCs and improve the delivery of drugs to these cells is needed. The development of such strategies holds great promise for the treatment of GBM and other cancer types that are characterized by chemo- and radiation resistance [95].

### 3.5. Intertumoral Heterogeneity

One of the biggest challenges in treating GBM is intertumoral heterogeneity, which refers to the fact that different regions within a single GBM tumor can have different molecular and genetic profiles. This heterogeneity makes it difficult to develop effective treatments that target all regions of the tumor. One reason for the intertumoral heterogeneity in GBM is the presence of cancer stem cells [96]. These stem cells are thought to be resistant to many forms of treatment, such as chemotherapy and radiation therapy, and may contribute to tumor recurrence and progression. Research has shown that GBM stem cells are heterogeneous in their molecular and genetic makeup, which contributes to the overall intertumoral heterogeneity in GBM [97]. In addition to the presence of cancer stem cells, intertumoral heterogeneity in GBM can also result from the unique microenvironment within the brain [98]. Despite these challenges, there have been recent advances in the understanding of intertumoral heterogeneity in GBM and the development of new treatments that aim to overcome this heterogeneity. The use of personalized medicine, which considers a patient’s specific molecular and genetic profile, has shown promise in the treatment of GBM. Additionally, the use of combination therapy, which involves using multiple treatments in conjunction with each other, is more effective than using a single treatment alone. However, much work remains to be conducted to fully understand the intertumoral heterogeneity in GBM and develop effective treatments that can target all regions of the tumor [99].

### 3.6. Intratumoral Heterogeneity

Glioblastoma multiforme (GBM) is a highly aggressive brain cancer with a poor prognosis and limited treatment options. One of the major challenges in treating GBM is the intratumoral heterogeneity, which refers to the fact that different cells within a single GBM tumor can have different molecular and genetic profiles. This heterogeneity makes it difficult to develop effective treatments that target all cells within the tumor. Intratumoral heterogeneity in GBM can result from multiple genetic and epigenetic changes, such as mutations, deletions, and amplifications of genes, which can occur during the evolution of the tumor [100]. These genetic and epigenetic changes can lead to the development of subpopulations of cells within the tumor that have different characteristics, such as drug resistance or increased invasiveness. As a result, it is difficult to develop a single treatment that is effective against all subpopulations of cells within the tumor. In addition to genetic and epigenetic changes, intratumoral heterogeneity in GBM can also result from the unique microenvironment within the brain. The brain is an immune-privileged site, meaning that it is protected from the immune system, and this can create a permissive environment for tumor growth and progression. The presence of immune cells, such as microglia and astrocytes, within the tumor microenvironment can also contribute to tumor resistance and progression [101]. Despite these challenges, recent advances in our understanding of intratumoral heterogeneity in GBM and the development of new treatments are offering hope for improving outcomes for patients with GBM. For example, the use of targeted therapies, which are designed to specifically target specific genetic alterations within the tumor, has shown promise in the treatment of GBM [102]. Additionally, the use of immunotherapy, which aims to enhance the immune response against the tumor, is a promising new treatment strategy for GBM. However, much work remains to be conducted to fully understand the intratumoral heterogeneity in GBM and develop effective treatments that can target all cells within the tumor.

### 3.7. Heterogeneity Caused by Extracellular Vesicles (EVs)

Glioblastoma multiforme (GBM) is a highly aggressive brain cancer that poses a significant challenge to effective treatment due to its heterogeneity. Recent studies have shown that extracellular vesicles (EVs) play a crucial role in driving this heterogeneity in GBM. EVs are small lipid vesicles that are released into the extracellular space by cells that can transfer biomolecules, such as proteins, RNA, and DNA, between cells.

One of the ways EVs contribute to GBM heterogeneity is by promoting the exchange of genetic and epigenetic information between cells within the tumor. For example, EVs can transfer genetic mutations from cancer cells to normal cells, leading to the development of new subpopulations of cells within the tumor with different genetic profiles [103]. Additionally, EVs can transfer RNA and DNA to recipient cells, which can result in changes in gene expression and regulation that can contribute to tumor progression and drug resistance [104,105]. Another way in which EVs contribute to GBM heterogeneity is by promoting the communication between cells within the tumor and the surrounding microenvironment. EVs can interact with immune cells, such as microglia and astrocytes, within the tumor microenvironment and modulate their behavior, leading to changes in the immune response against the tumor [106]. Furthermore, EVs can also interact with the extracellular matrix and modulate the physical properties of the tumor microenvironment, which can influence tumor cell behavior and progression [107]. Despite these challenges, the role of EVs in driving GBM heterogeneity is an area of active investigation and offers promising opportunities for the development of new therapies. For example, the use of drugs that target the release or the content of EVs has shown promise in preclinical models of GBM [108]. Additionally, the use of EVs as carriers for therapeutic agents, such as RNA and DNA, offers the potential for the development of new treatments that can target cells within the tumor in a more specific and effective manner [109].

## 4. New Therapies for the Treatment of GBM

Glioblastoma is a type of brain cancer that is one of the most aggressive and difficult to treat. The prognosis for patients with glioblastoma is typically poor, with a median survival time of only 15 months after diagnosis. New therapies for glioblastoma are important because they offer hope for improved outcomes for patients with this disease. Recent advances in the understanding of the molecular and genetic basis of glioblastoma have led to the development of new therapies that target specific genetic mutations and signaling pathways. For example, immunotherapy has shown promising results in clinical trials for glioblastoma, with some patients experiencing long-term survival after treatment. Other new therapies, such as those targeting the tumor blood supply and those using oncolytic viruses, are also under investigation and offer the potential for further improvement in patient outcomes. These new therapies have the potential to change the standard of care for glioblastoma and offer hope to patients and their families.

### 4.1. Immunotherapy

Cancer immunotherapy is a method of treatment involving an interference with the human immune system to increase or modify defense mechanisms against cancer. Immunotherapy can be divided into passive and active, each of which can be specific or non-specific. It is playing an important role in treating many types of cancers, and some therapies have also been approved for treatment by the US FDA [110,111]. However, successful treatment of GBM remains a major challenge. Immunotherapy seems to be one type of the treatments that can play an essential role in the GBM treatment in the future. There are various types of immunotherapy that have been applied to treat GBM such as CAR-T cell therapy, vaccines, immune checkpoint point inhibitors, and oncolytic viruses [111].

#### 4.1.1. Non-Specific Passive Immunotherapy

Non-specific passive immunotherapy involves the administration of agents or activated effector cells to non-specifically activate the immune system to produce an anticancer effect. This therapy can be carried out using, for example, cytokines or LAK cells (lymphokine activated killers). Cytokines are low-molecular-weight proteins that play an important role in all phases of the immune response, both humoral and cellular. To produce a biological effect, it is necessary to combine the cytokine with a specific receptor on target cells (T and B lymphocytes, natural killer cells, monocytes/macrophages, and granulocytes). Individual cytokines may have an antagonistic, agonistic, additive, or synergistic effect on the same biological processes. Anticancer effects of cytokines include the following:

- Direct cytotoxic effect (TNF-α);
- Modification of lymphocyte migration (TNF, IL-1, INF-γ) [112];
- Increased sensitivity of cancer cells to the cytotoxic effects of various biological or chemical agents (INF-γ, TNF-α) [113];
- Inhibition of tumor cell proliferation (INF-α, INF-γ);
- Activation of NK cells (GM-CSF, IL-2, IL-6) [112].

One such cytokine, interleukin-2 (IL-2), has shown promising results in clinical trials for the treatment of glioblastoma. IL-2 stimulates the growth and activity of immune cells, including T cells and natural killer cells, which can help to target and destroy cancer cells [114,115]. Another cytokine that has been investigated as a potential treatment for glioblastoma is interferon-beta (IFN-β). IFN-β has been shown to induce an anti-tumor immune response, as well as to directly inhibit the growth of cancer cells [116]. Recent findings show that developing next-generation cytokine therapies to achieve the long-awaited goal of harnessing their full therapeutic power to combat GBM might be the future of new immunotherapies. The combination of IL-12 and INF-α have shown some promising results in in vitro and in vivo studies [117].

#### 4.1.2. Specific Passive Immunotherapy

Specific passive immunotherapy is a treatment method based on the administration of factors or effector cells specifically directed against a given tumor cell. Examples include antibodies against antigens found on tumor cells, cellular therapies using tumor infiltrating lymphocytes (TIL) that are isolated, multiplied, activated, and then transfused again, or peripheral blood lymphocytes stimulated in vitro with antigen. Certain hopes are also pinned on therapy based on the modification of autologous lymphocytes isolated from peripheral blood (peripheral blood lymphocytes (PBLs) [118]. Modified specific mAbs used in immunotherapy act by directly binding to the tumor antigen and activating ADCC and complement-dependent cytotoxicity (CDC). They can also block receptors on cancer cells, for example, growth factors. Antibodies combined with a radioisotope, a cytostatic drug, enzymes, cytokines, or toxins directly kill the cells that are coated with them [119].

Antibodies are a type of immunotherapy being actively investigated as a treatment for glioblastoma multiforme (GBM). One example of an antibody that is being investigated as a treatment for GBM is bevacizumab. Bevacizumab is a monoclonal antibody that targets vascular endothelial growth factor (VEGF), a protein that stimulates the growth of blood vessels that feed tumors. In clinical trials, bevacizumab has been shown to have anti-tumor activity in GBM patients and to improve survival when used in combination with other treatments, such as radiation and chemotherapy [120].

Another antibody that is being investigated as a treatment for GBM is cetuximab. Cetuximab is a monoclonal antibody that targets the epidermal growth factor receptor (EGFR), a protein that is over-expressed in many types of cancer, including GBM. In preclinical studies, cetuximab has been shown to inhibit the growth of GBM cells and to enhance the efficacy of other treatments, such as radiation therapy [121,122].

An antibody that is also being investigated as a treatment for GBM is aflibercept. Aflibercept is a fusion protein that binds to VEGF and other growth factors that regulate blood vessel growth. In clinical trials, aflibercept has been shown to have anti-tumor activity in GBM patients and to improve survival when used in combination with other treatments, such as radiation and chemotherapy [123].

Atezolizumab is a monoclonal antibody that targets programmed death-ligand 1 (PD-L1), a protein that helps to suppress the immune response to cancer cells. In clinical trials, atezolizumab has been shown to have anti-tumor activity in GBM patients and to improve survival when used in combination with other treatments, such as radiation and chemotherapy [124]. Another antibody that also targets PD-L1 is pembrolizumab. In clinical trials, pembrolizumab has been shown to have anti-tumor activity in GBM patients and to improve survival when used in combination with other treatments, such as radiation and chemotherapy [125].

One of the most promising examples of immunotherapy for GBM is adoptive cell therapy. Chimeric antigen receptor T cells (CAR-T) genetically modify T-cells in the human body to specific targets by recognizing surface protein on the tumor cells. They can also be optimized to augment their binding and signaling properties [126]. Many specific targets have been identified. The most important ones include HER2, EphA2, EGFRvIII, and IL-13Rα2-CAR [127]. EGFRvIII is present in 52% of mutated glioma cells but absent in healthy tissues. During a clinical study, tumors underwent antigenic loss [128]. CAR-T-secreting BiTEs, which also contain EGFRvIII, have also been developed [129]. Brown et al. developed CAR-T for cytotoxic against IL-13Rα2^+^ cells, but they also showed antigenic loss [130]. Kim et al. improved control of CAR-T by developing Syn-Notch, which helps to differentiate tumor antigens precisely [131]. Additionally, CAR-Ts combining scorpion peptide with Chlorotoxin (CTLX) were developed to eradicate the tumor. CTLX, known for identifying glioma cells did not affect the healthy cells of patients. Clinical trials are in progress (NCT04214392). It will help CTLX-CAR-Ts to be effective in treating GBM. Other approaches include attaching helper genes [132]. Huang et al. developed CD70-CAR because CD-70 is highly expressed in glioma cells [133]. Bielamowicz et al., engineered a Trivalent CAR directed against IL-13Rα2, HER, and EphA2. It showed great efficacy in vivo and in vitro [134].

#### 4.1.3. Non-Specific Active Immunotherapy

Non-specific active immunotherapy is a treatment method that stimulates the immune system, especially the cellular response, with antigens that are not found in cancer cells. Historically, microbes, their fragments, enzymes, and hormones have been used. Recently, using genetic engineering technology and an increasing understanding of the immune mechanisms associated with cancer, mAbs modulating the immune response have been constructed. Substances that stimulate the immune processes include non-specific immunostimulators and immunomodulators. Herpes simplex virus type 1 (HSV-1) is a common virus that can infect and kill cancer cells. In preclinical studies, HSV-1 was effective against GBM cells and induced an immune response against cancer [135]. Ad-RTS-hIL-12 is a genetically modified adenovirus that is designed to release a therapeutic protein, interleukin-12 (IL-12), when activated by a drug. In preclinical studies, Ad-RTS-hIL-12 is effective against GBM cells and to induce an immune response against cancer [136]. Reovirus is a common virus that can infect and kill cancer cells. In preclinical studies, reovirus has been shown to be effective against GBM cells and to induce an immune response against cancer [137].

#### 4.1.4. Specific Active Immunotherapy

Specific active immunotherapy is a treatment method based on stimulating immunity to antigens specific to a given type of cancer.

Specific active immunotherapy includes immunization using the so-called therapeutic cancer vaccines. They include:

- Non-cellular vaccines—peptide, HSP (heat shock protein—vaccines based on heat shock proteins), DNA, and viral vaccines;
- Cellular vaccines—unmodified and genetically modified, and DC cells “fed” with tumor antigens.

For glioblastoma treatment, few types of vaccines have been tested. One of them is the autologous dendritic cell vaccine, which is made from a patient’s immune cells. The cells are collected, treated in the laboratory to make them more potent at stimulating an immune response, and then re-injected into the patient. Autologous dendritic cell vaccines are safe and well tolerated in clinical trials of GBM patients and induce an immune response against cancer [138,139].

Another type of vaccine is the tumor lysate vaccine: tumor lysate vaccines are made from pieces of a patient’s tumor that are treated in the laboratory to create a vaccine. Tumor lysate vaccines are safe and well tolerated in clinical trials of GBM patients, and induce an immune response against cancer [140].

The most promising vaccine is a personalized peptide vaccine made from small pieces of proteins that are specific to a patient’s cancer. The vaccines are designed to stimulate an immune response against these proteins and, in turn, against cancer. Personalized peptide vaccines have been shown to be safe and well tolerated in clinical trials of GBM patients, and to induce an immune response against the cancer [141].

### 4.2. Inhibitors

In recent decades, with advances in cellular and molecular biology, cancer treatment has undergone changes from non-specific cytotoxic to targeted drugs [142]. As the name says, targeted therapy targets specific proteins that control cell cycle, including their growth, division, and spreading [143]. Targeted drugs include small molecules, including tyrosine kinase inhibitors, and macromolecules, as monoclonal antibodies [142]. Despite the increasing number of target therapies for cancer approved by the FDA, only Bevacizumab (BVZ), a monoclonal antibody inhibitor of vascular endothelial growth factor (VEGF) protein, is approved for glioblastoma treatment [144,145]. However, some other treatments alone or in combination have been studied [38].

Tyrosine kinase (TK) proteins are a large, multigene family of proteins, whose function is related to the regulation of cell-to-cell signaling. They can be found in cell membranes or in the cytoplasm and are classified as TK receptors or non-receptors, respectively [146]. TK proteins play important roles in diseases such as cancer and diabetes, mostly due to their function as regulators of cellular processes and homeostasis [146,147]. More than 70 tyrosine kinase inhibitors (TKI) have already been approved and successfully used in clinical practice for several tumors, such as metastatic breast cancer, metastatic renal-cell carcinoma, advanced hepatocellular carcinoma, chronic myeloid leukemia, and gastrointestinal stroma tumor [148]. GBM presents mutations and regulation issues in several tyrosine kinase receptors, including platelet-derived growth factor (PDGF), vascular endothelial growth factor receptor (VEGFR), fibroblast growth factor receptor (FGFR), and epidermal growth factor receptor (EGFR), which are attractive and studied as targets for therapy in clinical studies [38,149,150,151].

Inhibitors of EGFR, such as Osimertinib, Lazertinib, Erlotinib, Gefitinib and Afatinib, have shown potential results for GBM in in vitro and in vivo mouse models. However, clinical outcomes have not been so encouraging [146]. Even though good tolerability was reported, therapy with Gefitinib failed for most participants in the first 8-week evaluation of a phase II clinical trial for recurrent GBM [152]. Previous benefits shown in preclinical studies were also not confirmed in clinical trials with Erlotinib, as monotherapy or in combination [38,146].

VEGF inhibitors have also been studied in recurrent GBM. An example includes Cediranib, a small molecule pan-VEGF receptor inhibitor, which has the advantage of oral administration with once-daily dosing [153] but has not received FDA approval. In a phase III trial, Cediranib was evaluated as a monotherapy and in combination with Lomustine (an alkylating agent) and failed to show a significant difference in progression-free survival or overall survival when compared to Lomustine alone [154]. Other anti-VEGF TKIs with less specificity, including Vatalanib, Pazopanib, Cabozantinib, and Vandetanib, have shown limited results in phase II trials [155].

Imatinib, Dasatinib, Sorafenib, Sunitinib, and Cabozantinib are examples of multi-target TKIs that have been evaluated for GBM [38,146,150,151], even though, until now, no TKI has been approved for GBM, mostly due to lack of success in clinical trials. This was attributed to their restricted delivery across blood–brain barrier and lack of tumor specificity [146,151].

Another potential target for inhibitors is poly (ADP-ribose) polymerase (PARPs), a family of intracellular enzymes that play important roles in DNA repair [156,157]. PARP inhibitors that block this DNA repair pathway, work especially well when other repair mechanisms are also impaired [158]. Preclinical studies with PARP inhibitors in GBM showed encouraging results, such as improved radiosensitivity in cells, effectiveness in sensitizing irinotecan- and temozolomide-resistant tissues, reduced growth of GBM cells, and improved survival in animal models [159]. Olaparib, Niraparib, Talazoparib, Veliparib, and Pamiparib are PARP inhibitors under clinical trials for GBM, being evaluated in combination with radiotherapy, chemotherapy, antiangiogenic therapies, and immunotherapy [158,159]. Despite many efforts, the benefits of PARP inhibitors in GBM clinical trials remain to be determined [38], which could be attributed to limited blood–brain barrier permeation, tumor resistance to PARP inhibitors, and increased hematological toxicity when associated with temozolomide [158].

Integrins are involved in most resistance mechanisms of GBM, becoming a potential therapeutic target, with several completed and ongoing clinical trials [160,161]. Integrins are a type of transmembrane receptor and cell adhesion molecules composed of two subunits, α and β. They are involved in cell–cell and cell-extracellular matrix adhesion, playing an important role in cell signaling and in regulating cell proliferation, migration, and survival [160]. Cilengitide, for example, is a selective inhibitor of αvβ3 and αvβ5 integrins, which have been studied alone or in combination with other treatments, such as radiation therapy and temozolomide chemoradiotherapy. While early studies of cilengitide in GBM showed promise, larger clinical trials have not shown significant benefit. As a result, cilengitide is not currently approved for the treatment of GBM [162]. The same happened with Volociximab, a monoclonal antibody against human α5β1 integrin [163]. Both failures to achieve clinical translation could be associated with delivery-related issues, and some authors are working on including cilengitide in nanocarriers to overcome those issues [164].

Finally, mTOR (mammalian target of rapamycin) has also proved to be involved in GBM growth and progression [165]. Various mTOR inhibitors exhibited good responses in preclinical studies [166]. Everolimus and Vensirolimus are mTOR inhibitors that have been clinically evaluated for GBM therapy. However, until now, they have not shown improvements in imaging exams, either in outcomes as progression-free survival when used as monotherapy, or in added benefits in combination with bevacizumab [38,166].

Despite promising preclinical data, most of the inhibition therapy did not show beneficial effects in GBM clinical trials. This could be attributed to poor drug permeability through the blood–brain barrier and to the enrollment of patients without a molecular selection. Precision medicine protocols have now been evaluated, to determine the expression of molecular targets before treatment randomization. A study was conducted with 34 patients with recurrent GBM, grouped according to VEGF, EGFR, and phosphatase and tensin homolog (PTEN) expression. The study showed improved results when compared to studies lacking molecular selection and showed a response rate of 50% in the whole cohort [167].

### 4.3. Ketogenic Diet

Recent studies have suggested that a ketogenic diet (KD), which is high in fat and low in carbohydrates, may have potential as an adjuvant therapy for this disease. The theory behind this is that cancer cells rely heavily on glucose for their energy needs, and by restricting glucose intake through a ketogenic diet, the growth and survival of cancer cells may be impaired [168]. There is some evidence to support the use of a ketogenic diet in glioblastoma multiforme. One study found that patients who followed a ketogenic diet in addition to standard therapy had significantly longer progression-free survival and overall survival than those who did not follow the diet [169]. Another study found that a ketogenic diet may increase the effectiveness of certain chemotherapy drugs in treating glioblastoma multiforme [170].

Ketones have several positive effects on brain metabolism, including increased mitochondrial biogenesis, antioxidant activity, and epigenetic modulation of genes related to metabolism. Studies using animal models of glioma have demonstrated that the ketogenic diet can enhance the effects of radiotherapy, possess anti-inflammatory properties, and reduce peri-tumoral edema and angiogenesis [171]. The initial clinical evidence supporting the efficacy of KD in humans comes from its ability to control refractory seizures in both children and adults [172]. These studies suggest that the ketogenic diet may have therapeutic potential in treating glioblastoma, and further research is warranted to determine its optimal use in this context. Thus, several clinical trials are ongoing to assess the use of a ketogenic diet as an adjuvant therapy for glioblastoma patients as shown in Table 1 [173].

However, it should be noted that the use of a ketogenic diet in glioblastoma multiforme is still a relatively new and unproven approach, and more research is needed to fully understand its potential benefits and risks. Additionally, a ketogenic diet can be difficult to adhere to and may have adverse effects on quality of life, particularly in patients who are already dealing with the physical and emotional challenges of cancer treatment. As such, patients who are considering a ketogenic diet as a complementary therapy for glioblastoma multiforme should do so under the guidance of a qualified healthcare provider [174].

**Table 1:** The examples of ongoing clinical trials of the ketogenic diet for the treatment of glioblastoma multiforme [175].

NCT Number	Title	Conditions	Interventions	Outcome Measures	Phases	Last Update Posted
NCT01865162	Ketogenic Diet as Adjunctive Treatment in Refractory/End-stage Glioblastoma Multiforme: a Pilot Study	Glioblastoma Multiforme	ketogenic diet	To evaluate the safety of ketogenic diet as adjunctive treatment of treatment-refractory glioblastoma multiforme|To obtain pilot data on efficacy of ketogenic diet as adjunctive treatment of treatment-refractory glioblastoma multiforme|To evaluate tolerability of ketogenic diet as adjunctive treatment of treatment-refractory glioblastoma multiforme	Phase 1	9 November 2022
NCT02302235	Ketogenic Diet Treatment Adjunctive to Radiation and Chemotherapy in Glioblastoma Multiforme: a Pilot Study	Glioblastoma Multiforme of Brain	Ketogenic Diet Standardized Diet	Survival time|time to radiological (MRI) tumor progression|The incidence of treatment-emergent adverse events during treatment|Tolerability of ketogenic diet: rate of early discontinuation of subjects from the diet because of intolerability, defined as unwillingness by the subject to continue with the diet because of possible diet related side effects	Phase 2	11 August 2022
NCT02939378	Ketogenic Diet Adjunctive to Salvage Chemotherapy for Recurrent Glioblastoma: a Pilot Study	Glioblastoma Multiforme	Ketogenic diet Standard diet	Number of Participants with Treatment-emergent Adverse Effects|The Chemosensitivity of Tumor|Overall Survival|Ketosis|Quality of Life	Phase 1 Phase 2	20 October 2016
NCT05708352	A Phase 2 Study of the Ketogenic Diet vs. Standard Diet Guidance for Patients With Glioblastoma in Combination With Standard-of-care Treatment	Glioblastoma Multiforme	Behavioral: Keto Diet Behavioral: Usual Diet	Overall survival|Health-related quality of life 1|Health-related quality of life 2|Progression-free survival|Cognitive performance 1|Cognitive performance 2|Physical activity	Phase 2	2 February 2023
NCT04691960	A Pilot Study of Ketogenic Diet and Metformin in Glioblastoma: Feasibility and Metabolic Imaging	Glioblastoma	Ketogenic Diet Drug: Metformin	Ability to achieve and maintain ketosis|Tolerability of metformin	Phase 2	13 December 2022
NCT00575146	Ketogenic Diet for Recurrent Glioblastoma	Recurrent Glioblastoma	Dietary Supplement: TAVARLIN	Applicability as Measured by Discontinuation of Study Treatment Due to Intolerability|Progression-free survival|Overall Survival|Frequency of Seizures|Ketosis|Quality of Life	Phase 1	2 May 2014
NCT03451799	Ketogenic Diet in Combination With Standard-of-care Radiation and Temozolomide for Patients With Glioblastoma	GBM Glioblastoma	Ketogenic Diet Radiation: Standard-of-care radiation Drug: Standard-of-care Temozolomide	Safety of the intervention|Feasibility of the intervention|Overall Survival|Time-to-progression|Quality of Life (two months)|Quality of Life (four months)|Cognitive function (Hopkins Verbal Learning Test-Revised)|Cognitive function (Trail Making Test)|Cognitive function (Controlled Word Association Test)|Cognitive function (Montreal Cognitive Assessment)	Phase 1	13 January 2023
NCT03075514	Ketogenic Diets as an Adjuvant Therapy in Glioblastoma	Glioblastoma Glioblastoma Multiforme, Adult	MKD MCT	To assess retention and drop-out rates|Estimation of recruitment rates|Enrolment of patients Long-term retention|Dietary adjustments required to achieve ketosis|Self-reported dietary compliance|Calculated dietary compliance|MCT compliance|Ketosis levels|Dietetic time required for interventions|Protocol refinements required|Sample size estimates for future trials|Quality of life|Food acceptability|Gastrointestinal side effects|Changes to biochemical markers|Anthropometric changes|Completeness of data	Not Applicable	4 April 2019
NCT01754350	Calorie-restricted, Ketogenic Diet and Transient Fasting During Reirradiation for Patients With Recurrent Glioblastoma	Recurrent Glioblastoma	Calorie-restricted ketogenic diet and transient fasting standard nutrition	Progression-free-survival|Feasibility Measured as Median Number of Days on Diet Per Patient and Average Calorie and Carbohydrate Intake Per Day During Day 1–9|Safety and Tolerability as Defined as Number of Patients With Adverse Events|Overall Survival|Frequency of Seizures|Ketosis|Quality of Life as Measured by the EORTC Quality of Life Questionnaire|Depression|Attention|Response	Not Applicable	2 June 2021
NCT02046187	Ketogenic Diet With Radiation and Chemotherapy for Newly Diagnosed Glioblastoma	Glioblastoma (GBM)	Ketogenic Diet Radiation therapy Drug: Temozolomide	Number of participants with adverse events|overall survival|time to progression|quality of life	Phase 1 Phase 2	16 June 2021
NCT05183204	Paxalisib With a High Fat, Low Carb Diet and Metformin for Glioblastoma	Glioblastoma	Drug: Paxalisib Drug: Metformin Ketogenic Diet	Progression-free survival, defined as the survival rate at 6 months|Overall survival, defined as the time of first study treatment to death from any cause|Change in insulin levels|Change in tumor glucose uptake values	Phase 2	4 October 2022
NCT04730869	Metabolic Therapy Program In Conjunction With Standard Treatment For Glioblastoma Multiforme	Glioblastoma Multiforme	Standard Treatment Plus Metabolic Therapy Program	Mean daily blood glucose-to-ketone ratio during chemoradiation|Mean daily blood glucose-to-ketone ratio during adjuvant chemotherapy|Mean daily blood glucose-to-ketone ratio during the MTP, calculated separately on fasting and ketogenic diet days|Change in weight|Safety as measured by National Cancer Institute Common Terminology Criteria for Adverse Events (version 4)|Change in performance status as measured by Eastern Cooperative Oncology Group Performance Status scale|Change in leisure/exercise activity as measured by Godin Leisure-Time Exercise questionnaire|Change in quality of life as measured by Functional Assessment of Cancer Therapy—Brain questionnaire|Progression-free survival|Overall survival	Not Applicable	2 June 2021
NCT03278249	Feasibility Study of Modified Atkins Ketogenic Diet in the Treatment of Newly Diagnosed Malignant Glioma	Glioblastoma	Modified Atkins Ketogenic Diet	Assessment of inducing ketosis|Assessment of progression-free survival|Assessment of survival	Not Applicable	24 November 2021
NCT01535911	Pilot Study of a Metabolic Nutritional Therapy for the Management of Primary Brain Tumors	Glioblastoma	Energy restricted Ketogenic Diet (ERKD) (Metabolic Nutritional Therapy)	MRI imaging will be used to measure changes in brain tumor size.	Not Applicable	2 May 2022
NCT03160599	Restricted Calorie Ketogenic Diet as a Treatment in Malignant Tumors	Malignant Tumors	ketogenic diet	Adverse events of patients on high-fat diet	Not Applicable	5 September 2018
NCT02286167	Glioma Modified Atkins-based Diet in Patients With Glioblastoma	Glioblastoma Multiforme	Diet modification	Feasibility of intermittent modified Atkins diet in patients with GBM assessed by percent of patients able to remain on the diet and achieve nutritional goals|Biologic activity measured by pre- and post-study cerebral glutamate and glutamine concentrations assessed by MRS|Tolerability assessed by percent of patients who have an adverse reaction of any grade attributed to the diet of possible, probable, or definite|Dietary Activity	Not Applicable	12 May 2020

## 5. Nanocarrier Treatment Options

The modification of the nano delivery systems may enable the preparation of targeting and controlled release characteristics according to the pathological features of the tumor site. The treatment of GBM with a nano drug delivery system would not only improve the pharmacokinetic profile of drugs in the circulation, which may reduce the systemic toxicity and side effects of drugs, but also significantly increase the concentration of drugs. At present, many nano delivery systems have been developed for the treatment of GBM, including lipid systems, polymer systems, dendrimer systems, and metal systems [176].

### 5.1. Liposomes

Liposomes are a kind of widely used nano vesicle. Amphiphilic phospholipid molecules form bilayers and further assemble into spherical vesicles with hydrophilic lumens in the center. According to the hydrophilic and hydrophobic properties of the payloads, they are encapsulated in different positions of liposomes. A large number of studies have reported on the application of liposomes for central nervous system delivery [177]. The functionalization with ligands enhances the ability of liposomes to cross BBB and target GBM in the brain [178].

Six kinds of peptide-based ligands (Angiopep-2, T7, Peptide-22, RGDfK, D-SP5, and Pep-1), widely used in brain delivery system modification, were selected to conjugate to the surface of the liposomes, and their blood–brain barrier or blood–brain tumor barrier targeting capability was compared. Furthermore, in vivo imaging confirmed that c(RGDfK)/Pep-22-LP was more distributed in the brain than the single ligand modified liposomes [178]. The systematic study of the structure–activity relationship at the cellular level, was performed to compare transferrin and cell-penetrating peptide as a targeting moiety. Those moieties were attached to the liposomes by different lengths of the PEG chain (3.4 K) and PEG (2.0 K). Targeted liposomes showed significantly higher accumulation in brain microvascular-endothelial cells and C6 cells, while avoiding the capture by normal cells [179]. A brain-targeted liposomal codelivery nano preparation loaded with honokiol and disulfiram/copper (CDX-LIPO) was employed for combination therapy for remodeling of the tumor immune microenvironment. The α7 nicotinic acetylcholine receptor (nAChRs)-binding peptide CDX was conjugated to the surface of the liposomes to “shoot three birds with one stone”, multi-targeting the glioma vessel endothelium, glioma cells, and tumor-associated macrophages that all overexpressed α7 nAChRs [180].

### 5.2. Polymer Micelles

Polymer micelles are a kind of stable spherical nanostructure assembled by amphiphilic block copolymers in an aqueous environment. Polymer micelles have been extensively studied as nano carriers to deliver chemical drugs, gene drugs, protein drugs, and antibodies. Similarly to the research on liposomes, the micelles were surface modified for responsive drug release and target-specific drug delivery [181,182]. For that reason, αvβ3 and αvβ5 integrins overexpressed on the endothelial cells of GBM, and cyclic-Arg-Gly-Asp (cRGD) peptide could be employed for targeting the tumor. A cRGD-surface-installed pH-sensitive micelles system loaded with epirubicin effective for treatment of GBM was developed. The micelles effectively suppressed the growth in an orthotopic GBM model by delivering high levels of epirubicin and quickly released the drug by responding to the low pH through a hydrazone-bond in the tumor tissue [183]. A micellar system with (cRGD) modification also was employed as a delivery vehicle for photothermal/photodynamic therapy (PTT/PDT). A c(RGDfk)-modified glycolipid-like micelle (cRGD-CSOSA) encapsulating indocyanine green (ICG) was developed for PDT/PTT with NIR irradiation through the dual-targeting neovascular endothelial cells and tumor cells by cRGD. Histological evaluation revealed a significant increase in the apoptosis of tumor cells in the cRGD-CSOSA/ICG treated group with NIR irradiation [184].

Based on the characteristics of GBM, a variety of responsive strategies have been developed for the controlled release of micellar delivery systems. The first strategy involved the release of the payload in response to the redox microenvironment. Accordingly, the level of glutathione in GBM cells is usually higher than that of extracellular fluid, and glutathione reacts with the disulfide bond which would then break the linkage of polymer, leading to the controllable release of the drugs loaded in the micelles. Based on this responsive strategy, researchers developed self-assembled nanoparticles that were glutathione-responsive drug release polymer micelles [185,186,187]. The second was the release of the drug in response to the pH in the microenvironment. The pH in GBM tumors is drastically lower than that of normal tissues, which enables the acid-sensitive molecules in the polymer to break the micelles responsively so that the nano delivery system burst to release the drugs carried in tumor cells in a controllable manner [188,189]. Thirdly, the release of the drug could be controlled by high-intensity focused ultrasound. According to this strategy, doxorubicin/perfluorooctyl bromide-loaded polymer nanoparticles were able to release doxorubicin at tumor sites in a controlled manner, effectively improving the concentration of chemical drugs at tumor sites [190].

Some receptors are highly expressed on both the surfaces of BBB and tumor cells at the same time, including low-density lipoprotein receptor (LDLRs) [191], LDLR-related protein 1 and 2 (LRP-1 and LRP-2) [192], transferrin receptor (TfR) [193], lactoferrin receptor (LfR) [194], insulin receptor (IR) [195], and glucose transporter (GLUT1) [196]. The existence of these receptors makes it possible to realize a “kill two birds with one stone” targeting strategy for glioblastoma in situ [197].

### 5.3. Magnetic Nanoparticles

Magnetic nanoparticles (MNPs) possess the potential to respond to external magnetic fields, so they are potentially excellent carriers for antitumor drug delivery and medical imaging. For the treatment of GBM, superparamagnetic iron oxide nanoparticles can be employed to transform the alternating magnetic field into heat, which would then induce an immunogenic reaction in the body and promote the apoptosis of tumor cells. Thermal-responsive MNP are an excellent nano carrier with dual functions, including hyperthermia and drug-loaded chemotherapy [198,199].

Fe_3_O_4_@Au was developed as an MRI contrast agent [200]. Then, cetuximab (C225)-encapsulated Fe_3_O_4_@Au magnetic nanoparticles were invented and studied for targeting magneto-photothermal therapy against glioma cells [201]. To realize the goal of a theranostic, hybrid chitosan–dextran superparamagnetic nanoparticles (CS-DX-SPIONs) were prepared. CS-DX-SPIONs demonstrated targeted cumulation features in GBM and high MR contrast-enhancing properties [202]. A magnetic field inducing hyperthermia was also a good way to eliminate cancer cells. Temozolomide was incorporated in a biomimetic lipid-based magnetic nano vector, which showed tumor-specific release of the encapsulated chemotherapeutic drug and a hyperthermia treatment effect under a magnetic field [203]. Attempts to target the tumor site by magnetic field were never stopped. A nano-graphene oxide sheet functionalized with magnetic poly (lactic-co-glycolic acid) was prepared and used for glioma targeted delivery of radiosensitizing 5-iodo-2-deoxyuridine [204].

Retrospective clinical research suggested a high occurrence of resistance of GBM to anti-angiogenesis treatment. The tumor microenvironment (TME) of an anoxic, acidic condition was responsible for the transient effect of treatment. Hence, the remodulation of TME may be the key to solving the problem. To overcome treatment resistance and improve the effect of anti-angiogenesis therapy, a RGD-modified dual-valence manganese nanoparticle was synthesized, which also could work as an MRI contrast agent [205].

### 5.4. Other Nano-Complex Applications

Immunotherapy refers to a therapeutic method that uses drugs or biological agents to regulate the immune state of the body, so that the body activates an appropriate immune response to disease. There are many methods of tumor immunotherapy, including monoclonal antibody therapy, immune checkpoint blocker therapy, adoptive cell therapy, oncolytic virus therapy, and tumor vaccine, and immunotherapy based on a nanodrug delivery system is one of the most prominent [206,207]. In order to achieve sustainable T cell infiltration, a hydrogel comprising a tumor-homing immune nano regulator has been developed that induces immunogenic cell death and suppression of indoleamine 2,3-dioxygenase-1 and chemotactic CXC chemokine ligand 10. When delivered in the resected tumor cavity, the hydrogel system mimics a ‘hot’ tumor-immunity niche for attacking residual tumor cells and significantly inhibits postoperative recurrence of GBM [208]. Nanodiamond-polyglycerol-doxorubicin composites (Nano-DOX) were used to induce the onset of damage-associated molecular patterns (DAMPs), which could then effectively subvert tumor-associated immunosuppression and activate immunogenic cell death and active immunocytes, released from GBM [209,210].

Dendrimers were employed as a vector for chemical and gene agents for treating GBM with a huge loading capacity and a great potential for surface modification. PAMAM, as one of the most studied dendrimers, was engineered as a carrier for the delivery of antitumor drugs to GBM [211]. Amphiphilic peptide dendrimers [212], poly(propyleneimine) [213] and Poly(L-lysine) [214] were used for the same purpose as well. Surface modifications of dendrimers were studied for effectiveness in targeting GBM [215].

### 5.5. Nanocarriers in Clinical Trials for the Treatment of GBM

Although there have been significant advancements in understanding glioma pathogenesis and developing potential therapeutic strategies, glioblastoma remains an incurable disease with the lowest median overall survival (OS) rate among all malignant brain tumors. This is because the blood–brain barrier restricts the entry of most antitumor agents, and efflux pumps actively remove drugs out of the central nervous system, making it difficult to develop effective therapies. Nanomedicine offers a promising alternative for efficient brain drug delivery, as it can improve drug availability in a targeted and concentrated manner, potentially leading to lower drug doses and fewer side effects. Several clinically relevant drug candidates, including TMZ, nitrosoureas, platinum agents, integrin inhibitors, EGFR inhibitors, VEGFR inhibitors, HDAC inhibitors, topoisomerase inhibitors, and doxorubicin, have been loaded into nanocarriers and tested in orthotopic animal models of GBM [164,216]. The most widely studied drug to date for encapsulation into nanocarriers has been doxorubicin, under the assumption that overcoming its pharmacokinetic caveats could greatly enhance its therapeutic index. Different types of nanocarriers have been developed, including liposomal formulations and polymer nanoparticles, targeted across the BBB actively through binding to distinct BBB-targeting and/or glioma-targeting moieties, such as transferrin, angiopep-2, or anti-CD133 antibody, or through magnetic targeting for those inorganic nanoparticles made of magnetic-responsive materials. The inclusion of the drug candidates into targeted nanocarriers significantly prolonged the survival times of orthotopic rodent models of GBM. As a result of extensive effort in preclinical studies of nanomedicine approaches and their promising results, several nanocarriers have entered the clinical trials stage (Table 2) for the treatment of malignant glioma [164].

## 6. Future Treatment Perspectives

Glioblastoma, GBM, is an aggressive, hard-to-treat brain disease with a low rate of survival. Targeted therapy approaches are required to treat GBM for enhanced therapy outcome and prolonged survival of GBM patients. siRNA therapy may be effective for GBM treatment. Targeted therapy with siRNA drugs is effective for silencing GBM targeting genes or signaling pathways. However, siRNA therapy for GBM is hindered by several pitfalls including immunogenicity, low cellular uptake, inadequate blood circulation, poor blood stability, and weak penetration of the blood–brain barrier. The angiopep-2 (An2)-functionalized STST3siRNA-loaded exosomes (Exo-An2-siRNA) are a potential drug to enhance GBM treatment. Exo-An2-siRNA demonstrated excellent blood stability, enhanced cellular uptake, and effective BBB penetration ability. Exo-An2-siRNA exhibited an enhanced in vitro anti-GBM effect as exosomes protect siRNA and modified An2 target GBM effectively. Exo-An2-siRNA showed inhibition of proliferation in GBM in a U87MG xenograft model with no side effects and enhanced median survival time. This developed nanoplatform Exo-An2-siRNA is a promising, safe therapy approach for GBM. Designing a suitable siRNA drug delivery platform may be an effective therapeutic approach against GBM to get around the BBB, to enhance delivery inhibiting the deadly GBM and resulting in improved survival of GBM patients [218].

**Figure 3:**
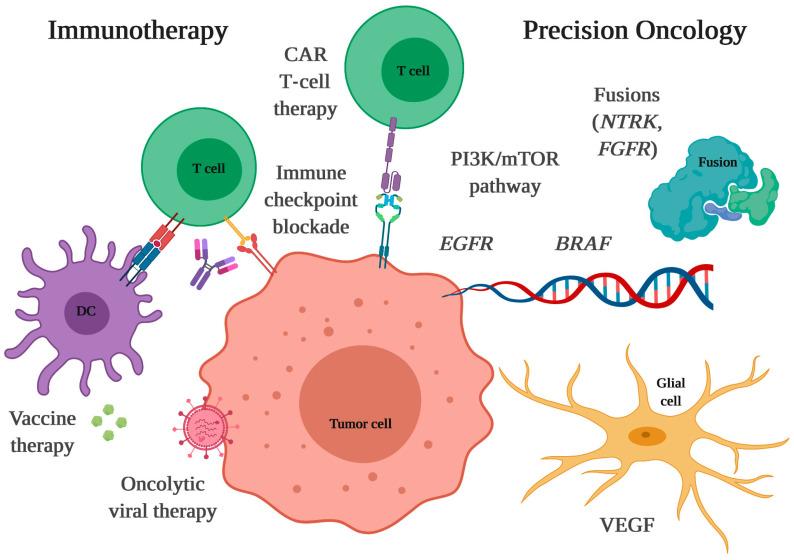
Immunotherapy and precision therapy approaches of GBM tumors. (Adapted with permission from Ref. [219]. 2020 American Cancer Society).

Precision therapy and immunotherapy may be more effective, tolerable, and promising therapies for undruggable GBM treatment (Figure 3) [219]. Immunotherapy approaches targeting immune checkpoint inhibitors such as PD-1/PD-L1 and CTLA-4 have been ongoing in GBM treatment. Further, clinical investigations are crucial for the improvement of immunotherapy of GBM. A combination of immunotherapy such as atezolizumab with chemotherapy may be a promising approach for GBM patients. Immunotherapy as well as EGFR-targeted therapies with TKIs have been extensively utilized for GBM with better consequence. The PI3K/mTOR signaling pathway is generally dysregulated in GBM with frequent *PTEN* loss, *PIK3CA* or *PIK3R1* mutations. More innovative clinical trials are crucial for utilizing biomarkers/targeting agents, and signaling pathways for the development of novel therapeutic strategies for GMB patients that reduce mortality [220]. Further, CAR-T cell therapy enhanced the response rate to GBM therapy, which is undergoing rapidly expansion for GBM patients. Targeted therapy, immunotherapy, and precision therapy utilizing CAR-T cells present prospective therapy approaches for GBM patients with enhanced survival.

The precision medicine approach for GBM may be highly promising (Figure 3). Next-generation sequencing technology has offered a better realization of the molecular mechanism and genomic architecture of GBM to detect targetable and actionable drivers of GBM tumors. Providing improved molecular profiling of GBM tumors is necessary for optimal patient management opportunities and state-of-the-art future direction of treatment [219].

GBM is a difficult-to-treat brain cancer and responds poorly to current therapies. Anti-VEGF antibody bevacizumab as monotherapy showed ~15 months survival. A combination of antidepressant imipramine, an autophagy enhancer, and VEGF inhibitor, bevacizumab indicated remarkable synergies in GBM therapy, co-targeting distinctive tumor-promoting mechanisms along with autophagy. This therapy remodeled robust GBM tumor vasculature to improve therapy outcome. In a mouse model of GBM, bevacizumab failed to improve survival. However, combining the anti-VEGF antibody with imipramine significantly delayed tumor growth in mice. Similar results were obtained utilizing small molecule TKI, axitinib, to inhibit VEGFR1-3. Thus, a combination of antidepressant drugs with therapeutics that inhibit VEGF-VEGFR signaling may be effective against GBM. The efficiency of combination therapy depends on IFN-γ signaling, which activates the expression of PD-L1 in tumors. The triple combination of antidepressant autophagy enhancer, VEGF inhibitor, and checkpoint inhibitor further enhanced the survival of GBM patients. These strategies are prospective therapy approaches for GBM [221].

In a healthy brain, the blood–brain barrier is a crucial part of the brain defense setup to prevent toxins, viruses, and bacteria from entering the brain. However, it may also prevent therapeutics from reaching the brain, causing a serious problem in treating conditions such as GBM or brain cancer. Recently, to overcome such difficulties, an implantable pump has been developed to bypass the blood–brain barrier to deliver chemotherapeutics to a specific brain area. Topotecan is effective against GBM. However, systemic delivery of topotecan is limited due to toxicity and insufficient brain penetration. An engineered subcutaneously implanted catheter pump to enhance delivery of topotecan into the brain was examined to evaluate its safety, efficacy, and biological effects in a patient with recurrent GBM, demonstrating a promising outcome. Thus, this innovative therapy approach utilizing an implantable pump for overcoming BBB could be an effective and emergent therapy option for GBM. However, further investigations are required to evaluate the toxicity and side effects of such implantable pump-based therapies as an approach for clinical applications [222].

Finally, timely diagnosis and early treatment initiation are crucial for GBM patients to improve therapy outcomes and survival. MRI has non-invasiveness and non-limited penetration depth and is a favorite tool for early GMB diagnosis. However, multimodal imaging in combination with MRI with other modalities synergistically integrates these results, overcoming the difficulties of each technique, and offering enhanced morphological and pathophysiological information on GBM tumors. Further, in addition to current MRI probes, double- or triple-modal nanoprobes may be utilized in multimodal imaging to improve imaging of the brain GBM sites to enhance GBM therapy and increase the survival of patients [223].

## 7. Conclusions

Chemotherapy, surgical resection, and radiation therapy are the major current treatment options for GBM. However, these therapy options are weak in effectiveness and have side effects. Therefore, overcoming the challenges in the development of advanced and effective therapies targeting biomarkers, new target agents, and dysregulated pathways are a promising approach. For example, EGFR-targeted therapies with TKIs have been extensively explored for GBM with a better outcome, VEGFR, PD-L1, and PI3K/mTOR pathway-targeted therapies may be promising either as monotherapy or in combination therapy. Further, innovative clinical trial design is crucial for the effective clinical translation of these therapies to improve the outcome in the patient reducing mortality. Targeted gene delivery to GBM siRNA drug delivery systems may be a potential therapeutic approach against GBM by overcoming the BBB, enhance delivery and improve survival. Targeted immunotherapy and precision medicine are beneficial to GBM patients. Recently, an implantable pump has been developed to bypass BBR to deliver chemotherapeutics effectively to specific brain areas in GBN tumors, which may be highly prospective for GBM patients. Furthermore, early diagnosis and fast treatment onset are significant for GBM patients to enhance therapy outcome and prolong survival. Multimodal imaging in a combination of MRI with other techniques is synergistic and an effective approach to reduce hurdles of each technique, thus offering a wealth of information on GBM tumors.

## Figures and Tables

**Table 2:** The ongoing clinical trials of nanocarriers for the treatment of glioblastoma multiforme [217].

NCT Number	Title	Conditions	Outcome Measures	Phases	Last Update Posted
NCT05768919	Study of Liposomal Curcumin in Combination With RT and TMZ in Patients With Newly Diagnosed High-Grade Gliomas	Glioblastoma	The number of observed Dose Limiting Toxicity (DLTs) The incidence of Adverse Events The proportion of patients at each dose level who receive at least 80% of the planned infusions of LC, 80% of RT, and 60% of TMZ during the first 10 weeks of treatment. Overall Survival (OS) Progression-free survival (PFS)	Phase 1 Phase 2	15 March 2023
NCT04573140	A Study of RNA-lipid Particle (RNA-LP) Vaccines for Newly Diagnosed Pediatric High-Grade Gliomas (pHGG) and Adult Glioblastoma (GBM)	Adult Glioblastoma	Manufacturing feasibility Safety of RNA-LP vaccine Determination of Maximum Tolerated Dose	Phase 1	21 February 2023
NCT01906385	Maximum Tolerated Dose, Safety, and Efficacy of Rhenium Nanoliposomes in Recurrent Glioma (ReSPECT)	Glioma	Phase 1: Maximum Tolerated Dose Phase 2: Overall Survival Phase 1: Dose Distribution Phase 1: Response rate Phase 1: Survival Phase 1: Safety of single dose of treatment Phase 2: Safety and tolerability of 186RNL Phase 2: Objective response rate (ORR) Phase 2: Progression-free survival at 6 months (PFS-6) Phase 2: Progression-free survival (PFS) from the initiation of study to first documented progression Phase 2: Quality of Life	Phase 1 Phase 2	12 December 2022
NCT05460507	Safety & Efficacy/Tolerability of Rhenium-186 NanoLiposomes (186RNL) for Patients Who Received a Prior 186RNL Treatment	Glioma	Assessment of safety and tolerability of a second dose of 186RNL by CED as part of standard of care > 30 days following first dose. Overall Survival Dose Distribution Overall Response Rate Progression-free survival	Phase 1	23 December 2022
NCT04881032	AGuIX Nanoparticles With Radiotherapy Plus Concomitant Temozolomide in the Treatment of Newly Diagnosed Glioblastoma	Glioblastoma	The recommended dose (phase I) of AGuIX in combination with TMZ and radiotherapy during the radio-chemotherapy period 6-month Progression-Free Survival (PFS) rate (phase II) Pharmacokinetic Cmax of AGuIX Pharmacokinetic Tmax of AGuIX Pharmacokinetic AUC of AGuIX Pharmacokinetic t1/2 of AGuIX distribution of AGuIX Overall Survival Progression-Free Survival (PFS) Toxicity (CTCAE criteria)	Phase 1 Phase 2	8 March 2023

## Data Availability

Data can be accessed from corresponding author upon request.
